# Tribological Properties of WS_2_ Thin Films Containing Graphite-like Carbon and Ni Interlayers

**DOI:** 10.3390/ma16010282

**Published:** 2022-12-28

**Authors:** Roman I. Romanov, Dmitry V. Fominski, Maxim V. Demin, Mariya D. Gritskevich, Natalia V. Doroshina, Valentyn S. Volkov, Vyacheslav Yu. Fominski

**Affiliations:** 1 National Research Nuclear University MEPhI (Moscow Engineering Physics Institute), Kashirskoe sh., 31, Moscow 115409, Russia; 2 Immanuel Kant Baltic Federal University, A. Nevskogo St 14, Kaliningrad 236016, Russia; 3 Center for Photonics and 2D Materials, Moscow Institute of Physics and Technology (MIPT), Dolgoprudny 141701, Russia

**Keywords:** ultralow friction, wear, nanolayered coatings, tribochemistry, WS_2_ thin films, graphitic-like thin films, pulsed laser deposition

## Abstract

The development and production of thin-film coatings having very low friction is an urgent problem of materials science. One of the most promising solutions is the fabrication of special nanocomposites containing transition-metal dichalcogenides and various carbon-based nanophases. This study aims to explore the influence of graphite-like carbon (g-C) and Ni interface layers on the tribological properties of thin WS_2_ films. Nanocrystalline WS_2_ films were created by reactive pulsed laser deposition (PLD) in H_2_S at 500 °C. Between the two WS_2_ nanolayers, g-C and Ni nanofilms were fabricated by PLD at 700 and 22 °C, respectively. Tribotesting was carried out in a nitrogen-enriched atmosphere by the reciprocal sliding of a steel counterbody under a relatively low load of 1 N. For single-layer WS_2_ films, the friction coefficient was ~0.04. The application of g-C films did not noticeably improve the tribological properties of WS_2_-based films. However, the application of thin films of g-C and Ni reduced the friction coefficient to 0.013, thus, approaching superlubricity. The island morphology of the Ni nanofilm ensured WS_2_ retention and altered the contact area between the counterbody and the film surface. The catalytic properties of nickel facilitated the introduction of S and H atoms into g-C. The sliding of WS_2_ nanoplates against an amorphous g-C(S, H) nanolayer caused a lower coefficient of friction than the relative sliding of WS_2_ nanoplates. The detected behavior of the prepared thin films suggests a new strategy of designing antifriction coatings for practical applications and highlights the ample opportunities of laser techniques in the formation of promising thin-film coatings.

## 1. Introduction

Scientific interest in thin-film coatings of pure transition-metal dichalcogenides (TMD) has been growing actively since the end of the last century. This holds especially for Mo/W/S_2/_Se_2,_ which have good antifriction properties when liquid lubricants are not applicable [1,2,3,4]. This interest has been drummed up by the rapid development of aerospace technology, vacuum technology, micro- and nano-electromechanical systems (MEMS, NEMS), and the search for alternatives to liquid lubricants used in weapons and metal machining—the latter inspired by environmental concerns [5,6,7,8]. Comprehensive studies of the morphology, structure, and chemical composition have revealed the main advantages and disadvantages of TMD coatings formed by ion-plasma and pulsed laser deposition (PLD) [9,10,11,12]. New avenues for modifying the composition and structure of such coatings are developing to overcome deficiencies such as low wear resistance, especially at high contact loads. TMD coatings have been doped with metal atoms (Ni, Ti, and others), as well as with carbon and nitrogen atoms [13,14,15,16,17]. Another approach to improving the tribological properties of TMD-based coatings is to create multilayer coatings in which TMD layers are alternated with layers of other TMD materials or carbon [18,19,20,21].

The latter strand of research has gained considerable momentum since the superlubricity effect is achieved in coatings formed from TMD and carbon nanolayers. When this effect is manifested, the friction coefficient does not exceed 0.01. The mechanisms behind this phenomenon, however, may differ. For this effect to occur, special interactions between the contacting surfaces are required at the atomic level. Incommensurate contact in the tribopair at an atomic level is considered a necessary condition for such an effect. The incommensurability can be achieved at the contact of surfaces with both ordered (crystalline) atomic packing and disordered local packing [22,23,24]. These conditions require an optimal structural state on the surface of the coating (in frictional interfaces) and the counterbody at the stage of friction pair preparation. A more promising and practical approach to achieving the superlubricity effect, which creates prerequisites for material self-organization in the contact area between the coating surface and the counterbody or between the coating layers. Triboinduced/tribochemical processes can initiate the formation of new states/phases in the contact area and, thus, reduce friction significantly. Tribochemical interaction between TMD and C-based components in the tribofilm can cause structural/chemical changes in both components. These alternations manifest themselves in the formation of new nanoparticles of different morphology, which change both the contact area in the friction pair and the friction mechanism [25,26,27,28].

The success of “constructing” new nanolayers depends on scientific advances in the understanding of triboadaptation mechanisms and the development of techniques for the formation of nanolayers based on TMD and carbon components. A combination of plasma-enhanced chemical vapor deposition (PECVD) and physical vapor deposition (PVD, ion-sputtering/magnetron deposition) techniques are commonly used to obtain such nanomaterials [18,29,30,31]. Sometimes, MoS_2_ flakes are deposited by drop-casting, ensuring the deposition of ultra-thin (2D/quasi-2D) MoS_2_ layers with the most perfect basal plane packing [32]. The PVD method helps to obtain TMD films whose nanocrystalline structure is usually amorphous or highly disordered. In this case, the conditions for ultralow friction are achieved under sufficiently high counterbody loads. The structural/chemical modification of the TMD layer is possible only under these conditions. Additionally, this also applies to the low shear force and incommensurability contact between adjacent TMD layers [29,31,32].

We investigated the formation of a nanolayer structure containing ultra-thin WS_2_ films, graphite-like carbon, and nickel, using pulsed laser deposition (PLD), including reactive PLD (RPLD). Studies in the field have mostly focused on MoS_2_-based films, whereas the application of WS_2_-based ultra-thin films to achieve superior lubrication with hierarchical/multilayer structures have not been treated in much detail. The well-known WS_2_ and MoS_2_ solid lubricants are extensively used materials today since they both have the most pronounced friction and wear reduced effect. The WS_2_ and MoS_2_ materials have both close-packed hexagonal laminar structure, and their crystal lattices contains thin layer units, which are formed by three plane layers of S, W, and S and S, Mo, and S, respectively. During friction, slippage develops easily along the closed packed plane. It is considered that MoS_2_-based coatings are soft; their antifriction properties are very good, but they have poorer corrosion/wear resistance, while WS_2_-based coatings are hard, and their corrosion/wear resistance and thermal stability are better. Formed during friction, WO_3_ is slightly more protective and provides a lower friction coefficient than MoO_3_ [19,33,34,35,36,37]. However, the generalizing characteristics are not universal, and deviations have been found in some experimental studies. Watanabe et al. [38,39] revealed that the friction-reduced properties of WS_2_ coating might be better than those of MoS_2_ coating.

Laser-based techniques open broad possibilities for the formation of ultra-thin TMD films with an adjustable number of basal planes and sufficiently perfect atomic packing [40,41,42]. The utility of PLD as a technique of obtaining graphite-like carbon films and metal films is well known [43,44]. However, interfacial solid-phase reactions can occur during the formation of multilayer films at high temperatures (up to 700 °C) ensuring the required characteristics of individual nanolayers. Further work is required to explore this phenomenon. A nickel film was used to initiate graphene-like phase formation in a carbon film [45,46]. We chose graphite-like carbon interlayers, which were obtained by vacuum PLD at an elevated temperature in the substrate. The deposition of carbon laser plasma at this condition provided sp^2^-bond formation in the g-C film, as well as the formation of W–C or Ni–C chemical bonds at the corresponding interfaces during the deposition of WS_2_/g–C/WS_2_ or WS_2_/g–C/Ni/WS_2_ thin-film coatings. It was also assumed that the local layered structure of g-C films can facilitate the penetration of heteroatoms from neighbor layers resulting in graphene-like structure formation during triboinduced processes.

Using RPLD/PLD, nanometer-thick films were created, consisting of one WS_2_ layer, three WS_2_/g-C/WS_2_ layers, and four WS_2_/g-C/Ni/WS_2_ layers. Tribotests were performed in an N_2_-enriched atmosphere, with a relatively low counterbody load. The superlubricity effect can be expected as a result of WS_2_ nanocrystals with basal atomic planes sliding along the surface of a g-C film modified by introducing heteroatoms. The retention of WS_2_ solid lubricating material on the surface of a carbon nanolayer can be achieved by the optimal disturbance of the smooth surface of the carbon layer.

## 2. Materials and Methods

**Figure 1:**
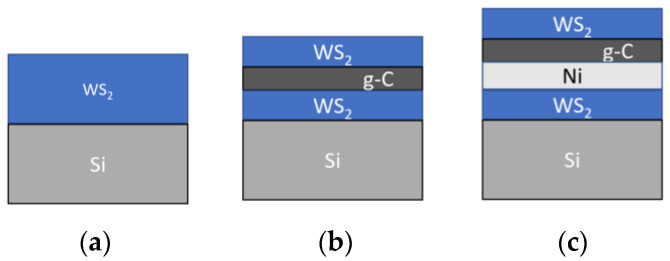
Schematic illustrations of thin-film coatings with different structures: (**a**) WS_2_, (**b**) WS_2_(C), and (**c**) WS_2_(C, Ni).

Layered thin-film coatings were created on polished silicon substrates in a single vacuum cycle. An Nd:YAG laser (LQ529, Solar LS, Minsk, Belarus) with a laser wavelength of 1064 nm, pulse duration of 15 ns, pulse energy of 40 mJ, and pulse repetition frequency of 20 Hz was used. The vacuum chamber was evacuated by a turbomolecular pump to a residual pressure of 10^−3^ Pa. WS_2_ nanometer-thick films were grown by RPLD. Using this technique, a WO_3_ target was irradiated in a reaction gas atmosphere (H_2_S) at 40 Pa. At the same time, the Si substrate was heated to a temperature of 500 °C. The deposition time was 30 s. During the deposition of nickel and carbon layers, targets of nickel foil and glass carbon plates were laser-irradiated in vacuum. The deposition time was 2 min and 1 min, respectively. The Ni layer was deposited at room temperature of the substrate; the g-C layer, at 700 °C. The thickness of all layers was 20–30 nm. In special cases (e.g., in MRS studies), the film thickness was increased to exclude the influence of the Si substrate signal on the measurements. In the following text, abbreviations will be used to refer to the coatings: WS_2_(C) for the three-layer WS_2_/g-C/WS_2_; WS_2_(C, Ni) for the four-layer WS_2_/g-C/Ni/WS_2_ (Figure 1). A scheme of fabrication of multilayer thin-film coatings using the laser technique is shown in Figure S1.

The morphology of sample surfaces and sample composition were studied by scanning electron microscopy (SEM) and energy-dispersive X-ray spectroscopy (EDS) using a Tescan LYRA3 device (Brno, Czech Republic). Structural analysis was carried out by X-ray diffraction (XRD) and micro-Raman spectroscopy (MRS). XRD measurements were carried out on an ARL X’tra diffractometer equipped with a parabolic mirror (Cu-K_α_ radiation was used). Measurements were performed in the Θ–2Θ geometry. Micro-Raman spectroscopy (MRS) provided insights into the detailed film structure. A LabRAM Evolution (Horiba Scientific, Kyoto, Japan) instrument with a 532 nm laser source with a 1 cm^−1^ spectral resolution was used to perform spectral measurements; the laser spot diameter was 0.45 µm. Laser intensity was kept under 0.2 mW to prevent laser-induced artefacts. Measurements were performed in a temperature-controlled room at standard conditions. The 520 cm^−1^ phonon mode from the silicon wafer was used for calibration.

The chemical state and composition of the films were analyzed by X-ray photoelectron spectroscopy (XPS), using a Theta Probe spectrometer under high-vacuum conditions (base pressure < 2 × 10^−9^ mbar) with a monochromatic Al-Kα X-ray source (1486.6 eV). Photoelectron spectra were acquired using the fixed analyzer transmission mode at 50 eV pass energy. For elemental composition XPS analysis, Scofield’s factors were employed in the calculations.

The tribological testing of thin-film coatings was carried out with the help of an Anton Paar TRB3 tribometer in the reciprocating motion mode, using a steel ball (100Cr6) with a diameter of 6 mm as a counterbody. The load on the ball was 1 N, and the Hertzian contact stress was ~660 MPa. The average speed of the ball over a coated substrate was 1 cm/s. The length of the wear track was 5 mm. The tests were carried out at a reduced atmospheric humidity (relative humidity RH ~ 8%), which was achieved by pumping N_2_ gas through the testing chamber. The sample temperature was 22 °C. The wear tracks and debris were studied by MRS, SEM, and optical microscopy.

## 3. Results

### 3.1. The Surface Morphology and Structure of the Films

**Figure 2:**
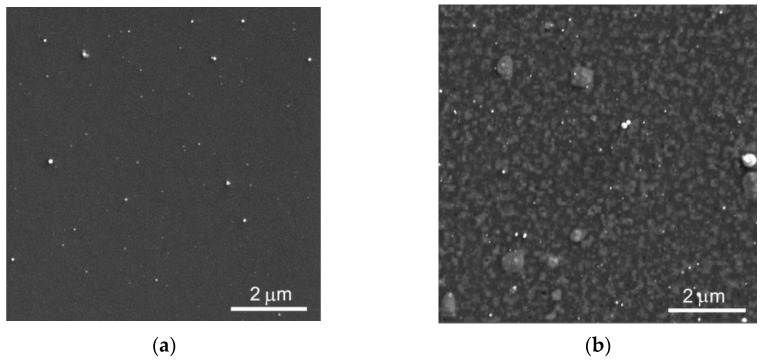
SEM surface images of (**a**) WS_2_(C) and (**b**) WS_2_(C, Ni) thin-film coatings on a Si substrate. The inhomogeneity of the Ni-containing film caused by the deposition of Ni droplets and the formation of Ni clusters on the surface of the WS_2_ underlayer (see Figure S2).

RPLD/PLD made it possible to form sufficiently smooth WS_2_ and carbon film on an area necessary for studying their tribological properties. Figure 2a shows the SEM image of a WS_2_(C) film before the tribotest. Rare round submicron particles were visible on the smooth surface of the film. These could be WO_x_ and carbon particles formed during the laser ablation of WO_3_ and glassy carbon target, respectively. Once within the track, such particles could cause abrasive wear and increase the coefficient of friction. The results of three friction coefficient measurements were used to analyze the tribological properties of the coatings.

Figure 2b shows that Ni film deposition is associated with marked changes in the surface morphology of the WS_2_(C, Ni) coating. These alterations were due to an increase in the surface concentration of micron-sized Ni particles increased during nickel deposition and the formation of a network of submicron-sized Ni clusters. Micron-sized particles were formed during the ablation of the Ni target and transported by the plasma plume to the coating surface. The surface concentration of microparticles was low, and of submicron particles, rather high. This difference was due to the particle formation mechanism. Coalescence and the growth of an island structure in the Ni layer were likely to occur during the deposition of the nickel atom flow onto the WS_2_ layer. These processes might have taken place when the bilayer Ni/WS_2_ film was heated before the carbon film deposition.

**Figure 3:**
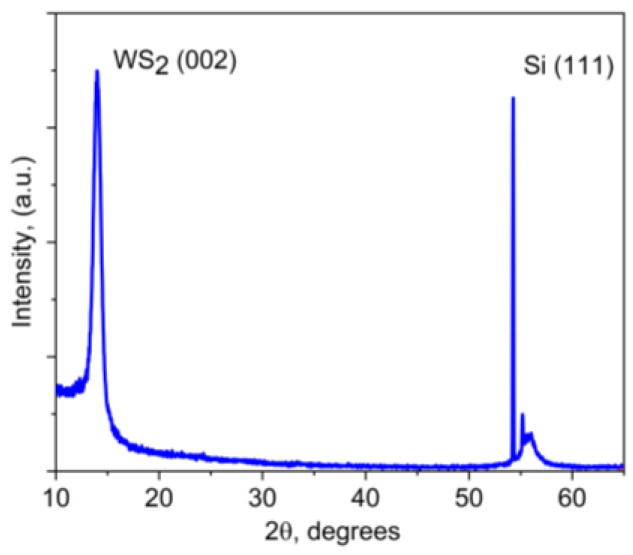
XRD pattern for the WS_2_ thin film obtained by RPLD on a Si substrate at 500 °C.

**Figure 4:**
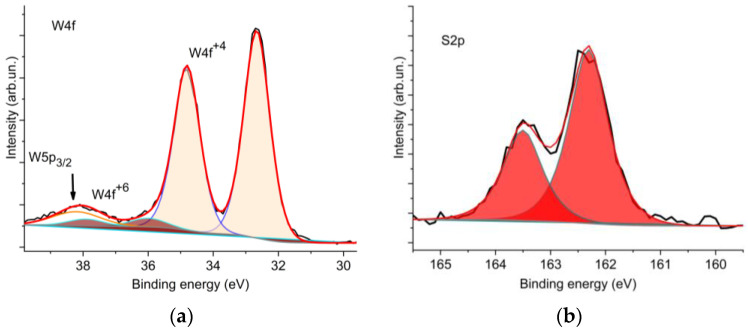
XPS spectra of (**a**) W4f and (**b**) S2p for the WS_2_ thin film obtained by RPLD at 500 °C.

**Figure 5:**
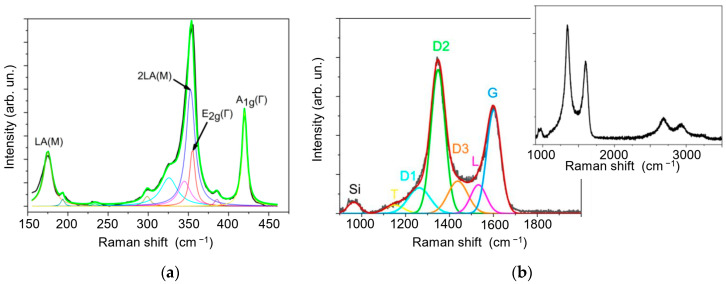
The MRS spectra of (**a**) WS_2_ and (**b**) g-C thin films obtained at 500 °C and 750 °C, respectively. The inset in (**b**) shows the MRS spectrum for a g-C film with second-order peaks typical of graphite-like carbon.

The data shown in Figure 3, Figure 4 and Figure 5a shed light on the characteristics of the single-layer WS_2_ film formed by RPLD on a Si substrate. The XRD pattern in Figure 3 shows not only the peaks of the silicon substrate but also an intense peak of the WS_2_ at 14°. This peak corresponds to basal planes (002) for the 2H-WS_2_ phase. This means that these basal planes mostly lay parallel to the substrate surface.

XPS spectra were analyzed to determine the chemical states of the WS_2_ films (Figure 4). The W4f spectrum was decomposed into two doublets. The principal doublet with W4f_5/2_ at 32.7 eV and a spin-orbit splitting of 2.1 eV corresponds to the state of W^4+^ in WS_2_. The weaker doublet with W4f_7/2_ at 36.0 eV and the same spin-orbit splitting value corresponds to the state of W^6+^ in WO_3_. In the compound, WO_3_ accounted for 5% at most. The S2p spectrum shows a doublet with S2p_3/2_ at 162.3 eV and a spin-orbit splitting of 1.3 eV, which corresponds to sulfur in WS_2_. As the figure shows, the selected RPLD mode ensures the effective sulfurization of tungsten and the removal of oxygen upon interaction between the deposited thin WO_x_ layer and hydrogen sulfide. The presence of a low-intensity peak of tungsten oxide in the XPS spectrum in Figure 4 is probably due to the introduction into the film of microparticles formed during the laser ablation of a WO_3_ target. The microparticles were not sulfurized completely.

Figure 5a shows the MRS spectra for a relatively thick WS_2_ film obtained by RPLD on a silicon substrate. The wavelength, 532 nm, for WS_2_ corresponds to the resonance of the exciton peak, C. Under these conditions, the obtained spectra contained first-order peaks (denoted as E_2g_^1^ (Γ) and A_1g_ (Γ)) corresponding to oscillatory modes inside the S-W-S layer in the parallel and perpendicular directions; overtones and combination peaks were also present. The most intense peak, with the center at about 352 cm^−1^, corresponds to the 2 LA(M) mode. There is also a peak at 175 cm^−1^, corresponding to the LA(M) mode. This first-order mode correlates with the acoustic phonon at the M point of the Brillouin zone. The relative intensity of this peak is used to assess the severity of defects in materials [47,48]. For microcrystalline WS_2_ films, the common type of defect is crystalline domain boundaries. Therefore, the relative intensity of the LA(M) peak correlates with the size of the domains, quite in accordance with earlier reports. In general, the spectrum of the film is typical of structures containing sub-µm grains [49,50]. The decomposition of the spectra was performed using the Lorentz function. The peaks were identified based on data from the literature.

Figure 5b shows the MRS spectrum for the g-C/Ni sample consisting of a g-C film deposited by PLD on a Si substrate with a Ni layer. This spectrum is characteristic of microcrystalline graphite [51]. The first order in the spectrum (1000–1800 cm^−1^) contains two well-defined peaks centered at about 1350 cm^−1^ and 1590 cm^−1^. In the literature, these peaks are referred to as the D and G lines. The G line corresponds to the doubly degenerate E_2g_ phonon mode at the center of the Brillouin zone. The position of this line is sensitive to changes in the length and direction of interatomic bonds in the grid of sp^2^-hybridized states. The position and FWHM of MRS peaks are usually taken as a measure of the disordering of the carbon network, present as distorted hexagonal rings and chains. The more defects in the structure, the smaller the Raman shift of the G line and the greater its width [52]. The D line appears in the presence of bond breaks. It corresponds to the A_1g_ phonon mode at the K point of the Brillouin zone. These lines can be used to estimate the size of crystal domains and the defectiveness of carbon films. The lateral size of the domains was estimated using the formula [53]: L_a_ = C(λ)I(G)/I(D), where C(λ) is the parameter determined by the wavelength of exciting laser radiation and I(D) and I(G) are the intensities of the D and G lines, respectively. The calculated L_a_ value was ~15 nm.

The spectrum was decomposed into Gaussian functions. The line centered at approximately 1150 cm^−1^ corresponds to trans-poly-acetylene chains [54]. The lines centered at about 1250 cm^−1^ (D1), 1350 cm^−1^ (D2), and 1440 cm^−1^ (D3) correspond to correlate with the breathing modes of benzene rings containing five, six, and seven atoms [55]. The line at 1530 cm^−1^ (L) is usually associated with vibrations in disordered carbon structures containing a certain number of sp^3^-hybridized states. It may be interpreted as a G line in amorphous diamond-like films [56,57]. The peak centered at 1590 cm^−1^ represents the G line for graphite.

**Figure 6:**
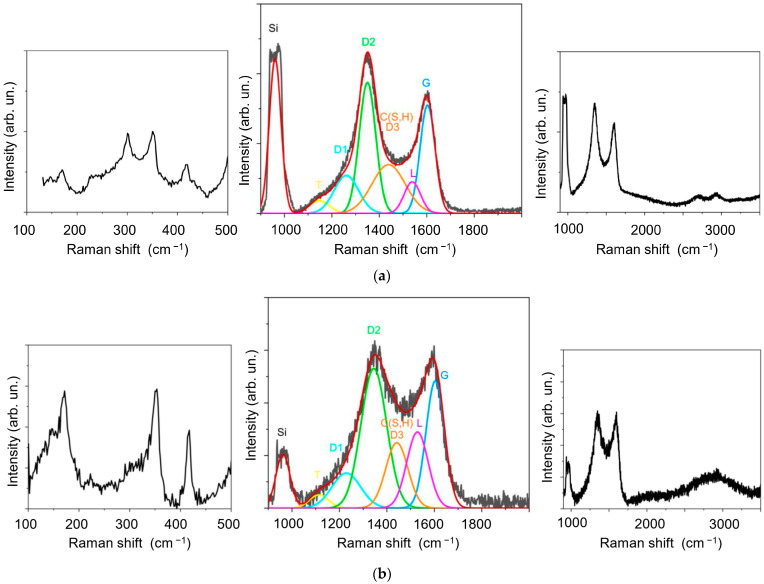
MRS spectra of g-C films for (**a**) g-C/WS_2_/Si and (**b**) WS_2_/g-C/WS_2_/Si samples. Insets show the MRS spectra for the WS_2_ (**left**) and g-C film (**right**), including second-order peaks.

As the WS_2_ film was deposited on the surface of the g-C film, the latter was exposed to laser plasma from a WO_3_ target, interacting chemically with the reaction gas H_2_S at a rather high temperature. This could change the chemical state of the interface between WS_2_ and g-C. Figure 6 shows the MRS spectra of the g-C/WS_2_ and WS_2_/g-C/WS_2_ samples (i.e., WS_2_(C)). The relative intensities of the D1, D3, and L lines increase in the decompositions of these spectra compared to the spectrum of the g-C/Ni sample (Figure 5b). The intensity of the peak at 2930 cm^−1^, which corresponds to the D + G mode, grew in the second order of the carbon spectrum. The ratio of the total intensities of the D1, D2, and D3 peaks to the G peak intensity (I(D)/I(G)) changed slightly (from 1.95 to 2.01 for all the samples). The FWHM of the G peak for the g-C/WS_2_ remained almost unchanged at 40 cm^−1^. Thus, it can be assumed that the size of crystallites does not change significantly, and the observed alterations in the spectra are due to the formation of lattice defects representing five- and seven-atom rings and amorphized regions.

These differences can be explained by the different influences of the underlayer. In the first case, it is a catalytic metal; in the second, WS_2_. Another possible cause is variations in the thickness of the g-C layer. It should be noted that the MRS peak at 1440 cm^−1^ may be of a different nature, namely, due to the formation of C(S, H) species following the introduction of sulfur and/or hydrogen into amorphous carbon. This was established by the authors of this study in earlier MRS studies of carbon films obtained by RPLD in hydrogen sulfide [58].

In the spectra decomposition for the g-C film in the WS_2_(C, Ni) sample, the intensity of lines D1, D3, and L grew even further (see Figure S3a). For instance, the ratio I(D3)/I(D) reached 0.32 compared to 0.15 for the g-C/Ni sample. The FWHM(G) value was as high as 60 cm^−1^. Another distinctive feature of the sample spectrum was the shift of the D3/C(H, S) peak from 1440 cm^−1^ to 1470 cm^−1^. The studies performed pointed out that the selected modes of forming a multilayer thin-film structure did not cause significant changes at the interfaces of the deposited nanolayers (e.g., Figure S3b). Yet, it can be assumed that the formation of the WS_2_(C, Ni) thin film coating was accompanied by modifications in the g-C layer under the influence of WS_2_ film deposition. This phenomenon manifested itself in changes in defectiveness and the possible introduction of S and H atoms into the surface of the g-C layer.

### 3.2. The Tribological Performance of the Obtained Films

**Figure 7:**
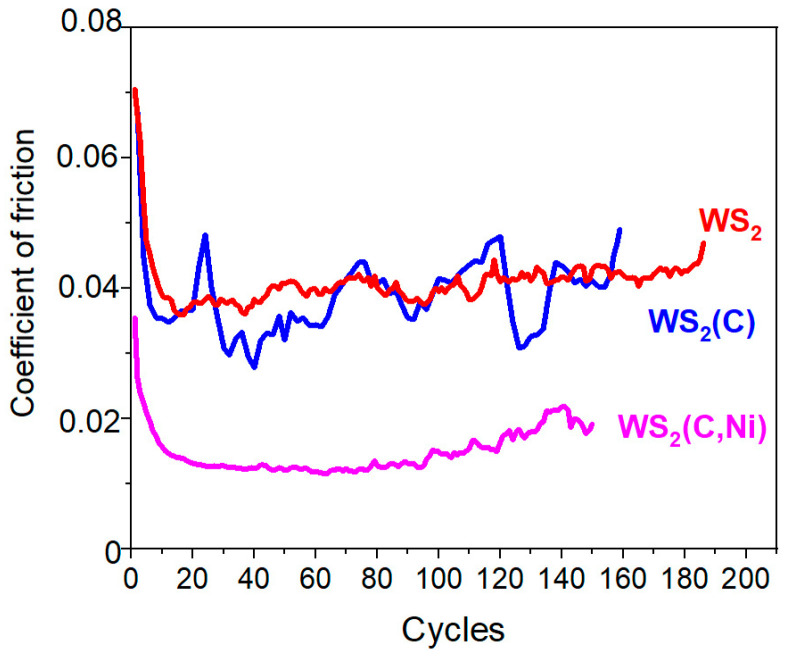
The characteristic evolution of the friction coefficient as a function of the number of cycles for different WS_2_-based thin-film coatings. For thin films, the tests were stopped as signs of a substantial increase in the coefficient of friction appeared.

Figure 7 shows the results of changes in the coefficient of friction when testing thin-film WS_2_, WS_2_(C), and WS_2_(C, Ni) coatings on a silicon substrate. The single-layer WS_2_ nanocoating withstood up to 200 cycles, and the coefficient of friction was approximately 0.04. The carbon interlayer had no significant effect on either the coefficient of friction or the durability of the WS_2_(C) coating. However, the sliding was accompanied by marked changes in the coefficient of friction. For the WS_2_(C, Ni) coating, the sliding of the counterbody occurred with minimum fluctuations in the coefficient, which decreased to 0.02 after running-in over several cycles and to 0.013 after 20 cycles. A rapid increase in the coefficient of friction was observed after ~130 cycles.

**Figure 8:**
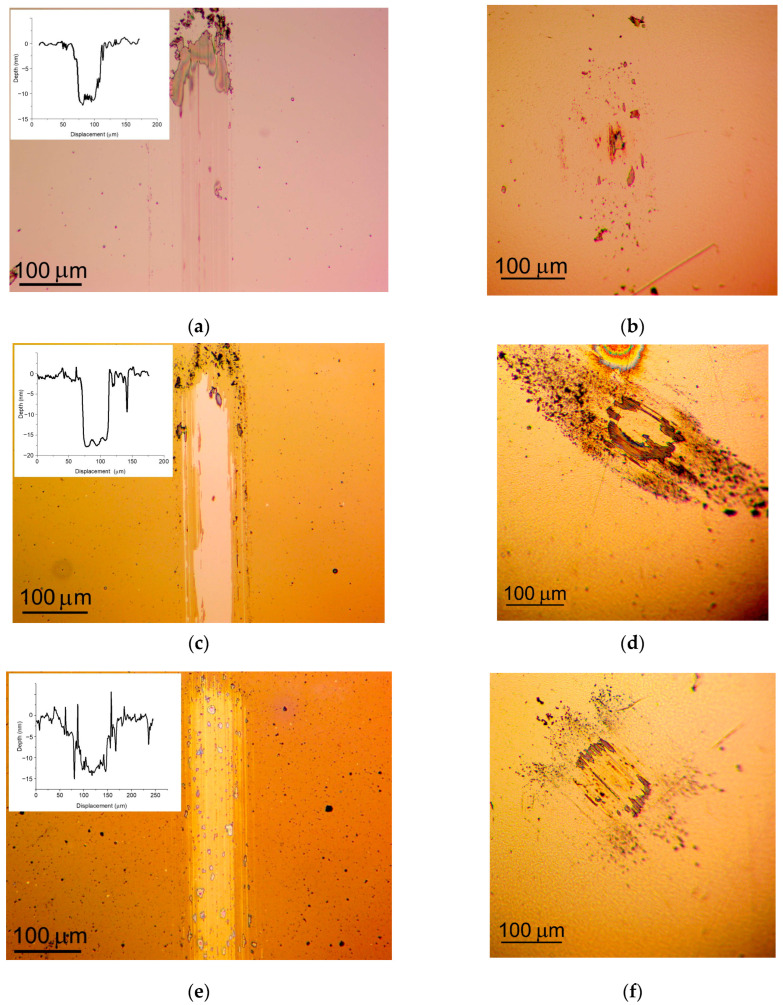
Optical images of the wear tracks (**left**) and the wear scars (**right**) on the coatings and steel balls, respectively: (**a**,**b**) WS_2,_ (**c**,**d**) WS_2_(C), and (**e**,**f**) WS_2_(C, Ni) thin-film coatings on a Si substrate. Figure 7 indicates test durations.

The coating composition had a significant effect on the wear pattern of the coating and the counterbody (Figure 8). When the WS_2_ coating was worn, wear debris effectively adhered as nanoplatelets to the coating surface at the counterbody reversal points. The wear of the counterbody was minimal. The WS_2_ coating wear plates barely adhered to the counterbody. The depth of the wear track was ~12 nm, and its width did not exceed 80 nm. The wear of the WS_2_(C) coating was a result of the effective adhesion of the wear debris on the surface of the counterbody. The depth of the wear track exceeded 17 nm, and the maximum width was 90 nm. During wear, the WS_2_(C, Ni) coating had the least amount of wear products, which adhered mainly to the counterbody. At the same time, the wear of the counterbody was most noticeable, which caused the wear track to widen to 90 nm. The depth of the track was approximately 14 nm.

### 3.3. Analysis of the Friction and Tribomodification of the Films

**Figure 9:**
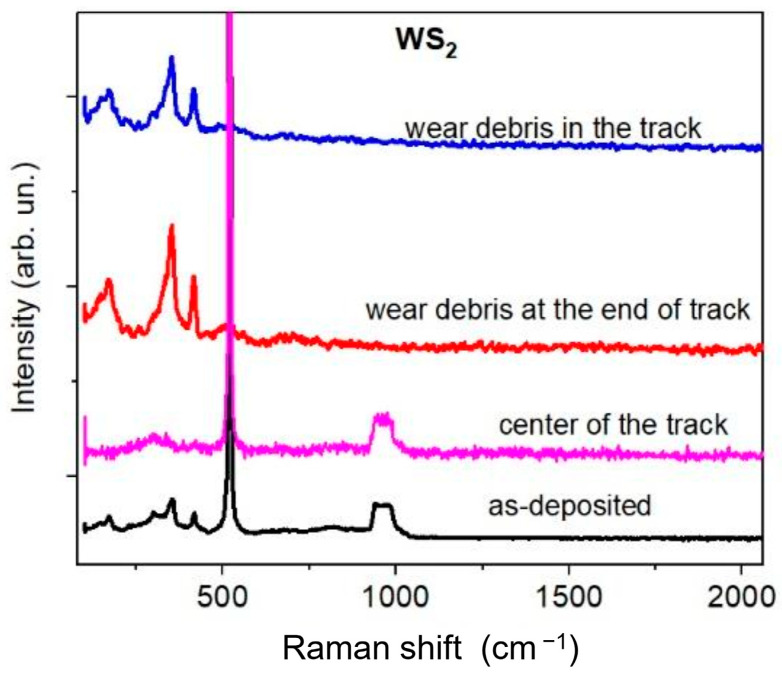
MRS spectra of the WS_2_ thin-film coating measured before and after tribotesting.

MRS spectra were measured inside the tracks at different points to analyze triboinduced changes in the coating structure. Wear debris located both inside the tracks and at the ends of the tracks (at the counterbody reversal points) was also examined. Figure 9 shows the results obtained for the thin-film WS_2_ coating. Although there was no coating in the center of the WS_2_ track, the coefficient of friction remained low. This could be due to a very thin tribofilm preserved in the track. The same film could remain on the surface of the counterbody. The MRS spectra for the wear debris localized both in the track and on its boundaries coincided with the spectra of the original film, i.e., had the structure characteristic of the crystalline WS_2_ phase. Thus, the MRS study showed that triboexposure does not cause appreciable changes in the structure of WS_2_ nanocoatings. When the counterbody was exposed, the WS_2_ nanolayers were removed layer-by-layer, accumulating mainly at the counterbody reversal points.

**Figure 10:**
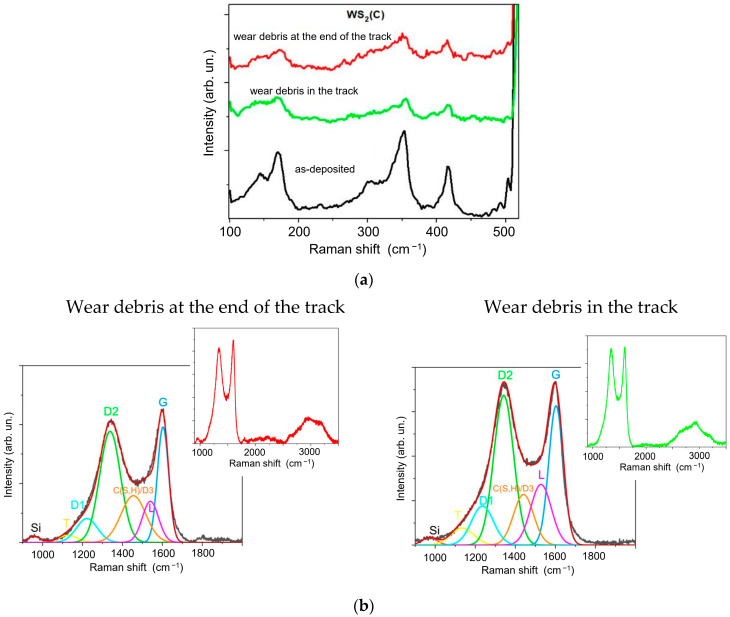
MRS spectra of the WS_2_(C) thin-film coating measured after tribotesting in (**a**) low- and (**b**) high-frequency intervals. The spectrum of as-deposited WS_2_(C) film is presented for comparison to illustrate the removement of WS_2_ film. The inserts in (**b**) show MRS spectra, which include second-order peaks. Spectra were measured for wear debris located at the end of the track and inside the track.

An MRS study of the WS_2_(C) thin-film coatings showed that the wear particles consisted predominantly of carbon: the peaks in the low-frequency region corresponding to WS_2_ had very low intensities (Figure 10). These peaks could be due to scattering on the WS_2_ film, which deposited on the silicon and remained under the wear debris. The intensity of the carbon spectrum, however, increased compared to the spectra of the original films. In the decomposition of the wear debris spectrum for the WS_2_(C) nanocoating, the relative intensities of lines D1, D3, and L lines diminished compared to the spectrum of the original sample. The I(D3)/I(D) ratio plummeted to 0.2, and the I(D)/I(G) ratio dipped from 1.95 to 1.85. The FWHM(G) value decreased to 40 cm^−1^. This led us to assume that tribotesting caused the dispersion of WS_2_ nanoplates in the wear debris of the carbon layer, which underwent only weak structural changes upon contact with the counterbody.

**Figure 11:**
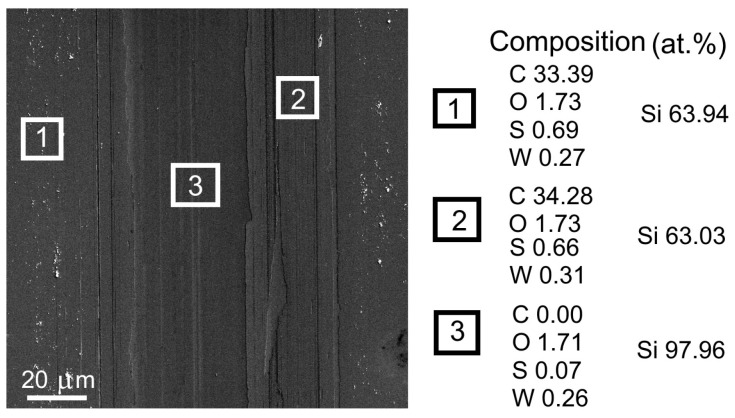
SEM image of the wear track formed after testing on the surface of the WS_2_(C)/Si sample. The composition was measured by EDS. Figure S4 shows the corresponding EDS spectra; Figure S5, the lateral distributions of elements across the wear track.

SEM and EDS studies confirm that the sliding of the counterbody when testing the WS_2_(C)/Si sample caused wear to spread across the carbon layer, which was almost completely removed towards the end of the test (Figure 11). Therefore, a wear mechanism is possible where the mechanical mixing of WS_2_ and g-C nanoparticles takes place. The dispersion of WS_2_ nanoparticles in the tribofilm was responsible for the relatively low coefficient of friction against the WS_2_(C) coasting, which is comparable to the coefficient of a single-layer WS_2_ coating.

**Figure 12:**
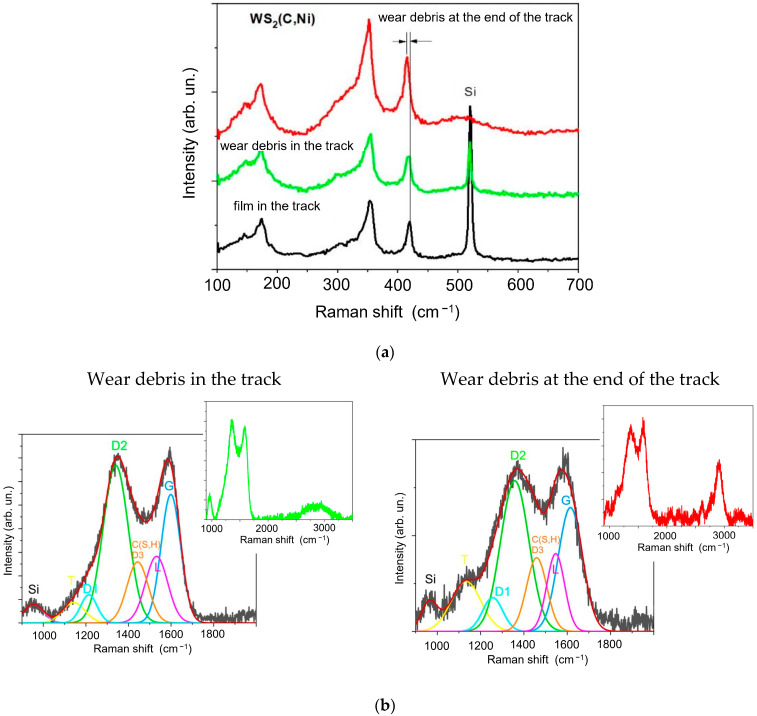
MRS spectra of the WS_2_(C, Ni)/Si sample after tribotesting in different regions of the wear track in (**a**) low- and (**b**) high-frequency intervals. MRS Si peak is shown for controlling the thickness of thin-film coating. The inserts in (**b**) show the MRS spectra, which include second-order peaks.

Figure 12 shows the MRS spectra measured for the WS_2_(C, Ni)/Si sample after tribotesting. Intense peaks corresponding to tungsten disulfide can be observed in the low-frequency region of the Raman spectra of the WS_2_(C, Ni) sample’s wear debris. The shape and relative intensity of these peaks are almost the same as in the spectra of the original films. For wear debris, the difference is that the position of the A_1g_ peak is shifted towards a decrease in the wave number by 1.7 cm^−1^. For ultrathin WS_2_ films, the shift between the E_2g_ and A_1g_ peaks is sensitive to the number of monolayers [59,60]. In this case, this shift changed from 65.3 cm^−1^ in the original film to 63.6 cm^−1^ in the wear debris. This difference corresponds to a decrease in the number of monolayers from about 10 to 5 [60]. Consequently, WS_2_ crystallites are ground into thinner flakes during the tribomodification.

For the WS_2_(C, Ni) sample, the carbon spectra of the wear particles in the track were slightly different from those of the original coating. Neither the I(D3)/I(D) nor the I(D)/I(G) ratio changed, remaining at 0.25 and 1.95, respectively. The FWHM(G) value (54 cm^−1^) did not alter either. The most significant changes were the shift of line D3 by 1440 cm^−1^ and a decrease in the I(D1)/I(D) ratio from 1.8 for the original sample to 1.1. Even more noticeable were the variations in the spectrum of the wear debris accumulated at the end of the wear track. The main change was the increase in the relative intensity of the peaks at ~1440 and ~2900 cm^−1^. According to the literature, the introduction of sulfur into exfoliated graphene and carbon nanotubes caused amorphization [61,62]. The annealing of the resulting nonequilibrium structures leads to the appearance of narrower bands with centers at ap-proximately 1250 and 1440 cm^−1^. According to [63], the annealing of graphene in sulfur vapor results in the appearance of lines with centers at 1436 and 1530 cm^−1^ in the Raman spectra. To confirm this, Raman spectra were measured using a higher intensity of exciting laser radiation (2 mW). The partial annealing of the WS2(C, Ni) films took place under these conditions. As a result, peaks with centers at approximately 1440 and 2890 cm^−1^ became clearly visible in the spectra (Figure S6). In addition, the increase in the relative intensity of line L may also be due to the formation of C-H bonds [64]. Hydrogen could be introduced into coatings during RPLD in hydrogen sulfide. Comparing the results of the MRS studies of triboinduced changes in WS2(C) and WS2(Ni, C) thin-film coatings suggests that the introduction of sulfur/hydrogen in the g-C layer is largely due to the catalytic effect of nickel.

**Figure 13:**
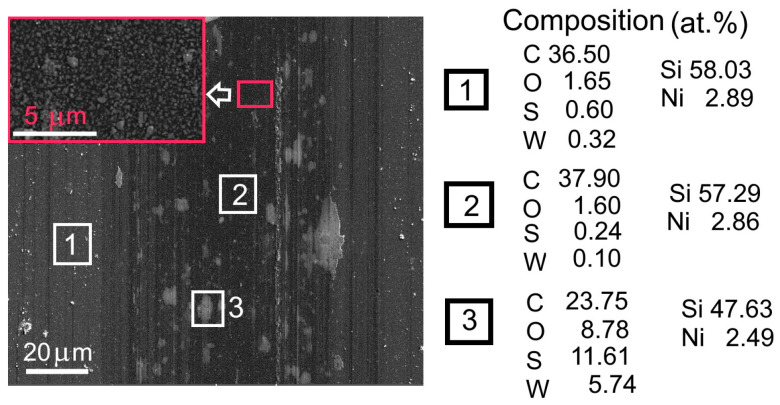
SEM image of the wear track formed after tribotesting on the WS_2_(C, Ni)/Si sample in the central region of the track. The EDS-measured compositions of the indicated areas near and inside the track are also shown.

**Figure 14:**
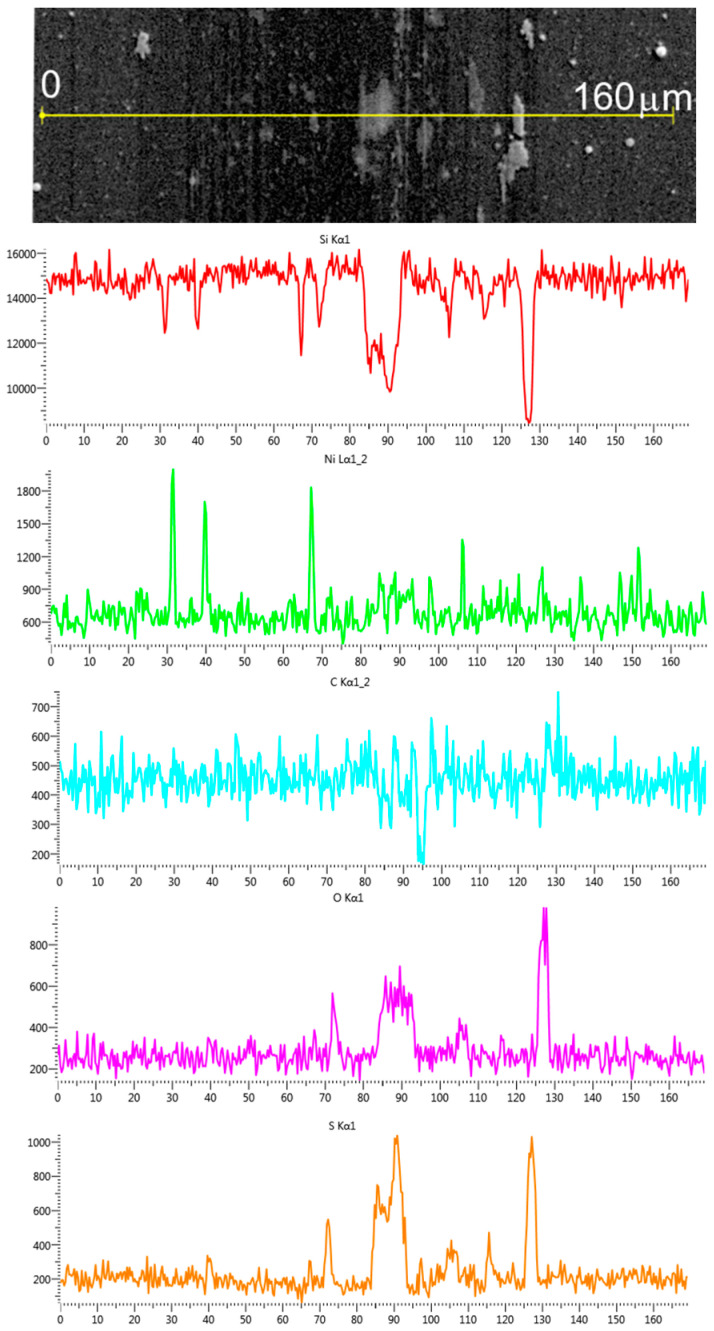
Distribution of different elements across the wear track for the WS_2_(C, Ni)/Si sample.

SEM and EDS studies of WS_2_(C, Ni) coatings on a Si substrate showed that tribointeraction disturbed the uniform distribution of WS_2_ in the track (Figure 13 and Figure 14). Figure S6 shows the EDS spectra for the selected area of this sample. Micro-regions were formed in the track in which WS_2_ accumulated. These regions appeared greyish in the SEM images. The distribution of elements in one of them is shown in Figure S7. An EDS study demonstrated that WS_2_-enriched areas were subject to partial oxidation. However, no appreciable peaks from WO_x_ were detected in the frequency range of 700–900 cm^−1^ in the sample’s MRS spectra. The presence of oxygen could be due to surface contamination. In the other sections of the track, the WS_2_ content decreased significantly. The carbon and nickel content barely changed, indicating that the tribomodification affected mainly the WS_2_ layer and the WS_2_/g-C interface. The nanostructured surface morphology of the Ni layer was preserved. The wear debris located in the track (125 µm coordinate) consisted mainly of C, O, S, and probably W. The study of tungsten by EDS in WS_2_-containing thin films deposited on a silicon substrate has its own deficiencies, which are due to the W Ma1 peak overlapping the intense Si Ka1 peak.

## 4. Discussion

The tribotests show that the single-layer WS_2_ nanocoatings are characterized by fairly good adhesion to the Si substrate. The coating consisted of 2H-WS_2_ nanocrystals having basal orientation. The perfect/non-defect basal plane of 2H-WS_2_ is characterized by weak chemical activity. However, during PLD, the surface of the Si substrate and the interface with the deposited WS_2_ film are bombarded by ions from the laser plasma. The ion energy may exceed 100 eV. Ion implantation could result in defect and new chemical bond formation on the surface of the underlayer and boost the adhesion of the deposited films. Our result correlates with the results of several studies, which found an improvement in the adhesion of WS_2_-based films to Si substrates and/or to pre-deposited W or Si-based interlayers (e.g., [35,65,66]. During friction, the nanocrystals could slide against each other in the near-surface layer. The wear occurred as a result of the displacement of WS_2_ surface nanoplates towards the ends of the tracks. The structure in the track and the wear debris almost did not differ from the original structure of the thin-film WS_2_ coating. This coating exhibited the highest durability.

The WS_2_(C) thin-film coating consisted of three rather smooth WS_2_/g-C/WS_2_ layers. A thin layer of graphite-like carbon doped with sulfur and/or hydrogen formed on the WS_2_/g-C interface. Probably, the sliding of the counterbody against the outer WS_2_ layer caused the WS_2_ nanoplates to slide against each other and the surface of the g-C film. In the case of weak layer adhesion at the g-C/WS2 interface, a mechanical mixture of WS_2_ and g-C nanoparticles could form on the surface of the underlying WS_2_ film. As the counterbody slid, the mixture of WS_2_ and g-C nanoparticles caused the coefficient of friction to fluctuate, which could cause the underlying WS_2_ film to crack and detach from the silicon substrate. The deep track profile with sharp edges pointed to this fracture mechanism. A thin g-C(S, H) layer was absent at the g-C/WS_2_ interface, which probably had an important influence on the friction and wear mechanism of the WS_2_(C) coating.

**Figure 15:**
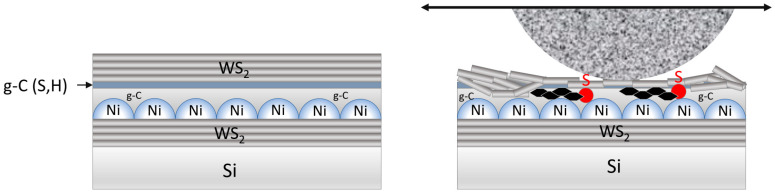
Schematic representation of the WS_2/_g-C/Ni/WS_2_/Si sample structure before (**left**) and during (**right**) contact with the counterbody during tribotests. Explanations are given in the text.

Figure 15 shows the morphology and structure of the prepared WS_2_(C, Ni) thin-film coating schematically. The deposition of the nanostructured Ni film caused the formation of irregularities on the coating surface. These irregularities, on the one hand, ensured the retention of the WS_2_ film on the surface of the WS_2/_g-C/Ni/WS_2_/Si sample. On the other hand, the irregularities contributed to the local pressure in the contact area between the counterbody and the coating. The sliding of the counterbody over the WS_2_ islands created the conditions for the permanent triboinduced transfer of the solid lubricant WS_2_ phase over the entire surface of the track. One might assume that the sliding of WS_2_ nanoplates against the surface of the interface g-C(S, H) layer creates the conditions for very low friction. There was no g-C(S, H) nanomaterial on the track due to wear accumulated at the ends of the track when the counterbody stopped. Remarkably, the doping of the g-C film surface with sulfur could continue as long as WS_2_ islands were present. During the triboinduced contact of the WS_2_ film with the g-C layer, sulfur atoms could penetrate the carbon, which probably contributed to the formation of new carbon forms, such as graphene-like carbon [25,55]. The efficiency of this process was probably controlled by the catalytic properties of nickel. The sliding of WS_2_ nanoplates having basal orientation against the amorphous g-C(S, H) layer with graphene-like nanophase inclusions could contribute to a very low friction coefficient. The conditions for very low friction disappear when the carbon layer is removed from the tops of the irregularities caused by the island-like nature of the Ni film structure and the more intensive wear of the counterbody begins.

It should be noted that the ultralow coefficient of friction for the WS_2_(C, Ni) thin-film coatings was due to the new realized coating architecture. Cao et al. [37] revealed that for nanocomposite WS_2_/a-C coatings when tested in low humidity conditions (5% RH), the friction coefficient was 0.021 at a counterbody load of 5 N. The work [37] contains a comparative analysis of the antifriction properties of WS_2_/a-C coatings with literature data on coatings of various compositions. For WS_2_/a-C coatings, ultrashort WS_2_ nanoplatelets were randomly distributed in an amorphous carbon matrix. The WS_2_ nanocrystallites form via selective atomic rearrangement from the amorphous bulk and join into longer crystallites because of defect climbing driven by frictional contact. It was shown that the single-layer WS_2_ coating we created provided a friction coefficient of ~0.04. Additional studies have shown that in the case of a higher load (5 N) on the counterbody, the coefficient of sliding friction over such a coating could decrease to ~0.02.

## 5. Conclusions

Multilayer thin-film coatings containing WS_2_, g-C, and Ni nanolayers were created using pulsed laser deposition. The structural and chemical state of these layers and their thickness (in the nanometer range) were controlled by PLD/RPLD modes and the deposition time. When sliding in a nitrogen atmosphere (RH~8%), a single-layer nanocoating consisting of 2H-WS_2_ nanocrystals having a basal orientation was subjected to wear by the mechanism of the layer-by-layer removal of the coating material. The coefficient of friction was ~0.04; it was determined by the resistance to the relative slip of the basal planes in the 2H-WS_2_ structure.

The alternation of WS_2_ and g-C nanolayers in the WS_2_(C) thin-film coating did not reduce the friction coefficient. Tribointeraction caused the formation of a mechanical mixture of WS_2_ and g-C nanoparticles, providing a coefficient of friction of ~0.04; the mixture was relatively quickly removed from the wear track. The incorporation of the Ni nanolayer into the WS_2_(C, Ni) structure caused the coefficient of friction to decrease to values approaching superlubricity. The lowest value of the friction coefficient in nitrogen with a counterbody load of 1 N was 0.013. This was due to the movement of WS_2_ nanoplates against the amorphous g-C layer doped with sulfur and/or hydrogen. S and/or H atoms could be introduced into the carbon layer from hydrogen sulfide (during the RPLD of a WS_2_ film on the g-C nanolayer) and as a result of the tribochemical reaction between WS_2_ and g-C. The decrease in the friction coefficient could be explained by the transformation of the graphite-like state of carbon into the graphene-like one under the catalytic effect of nickel. Nickel deposition was accompanied by the formation of submicron-sized (in plane) Ni particles, which changed the contact conditions between the counterbody and the thin-film WS_2_(C, Ni) coating and contributed to the ultralow coefficient of friction at a relatively low load (1 N) on the counterbody.

## Data Availability

Not applicable.

## Supplementary Materials

The following supporting information can be downloaded at: https://www.mdpi.com/article/10.3390/ma16010282/s1, Figure S1: A scheme of fabrication of multilayer thin-film coatings using the laser technique: (a) RPLD of thin WS_2_ film on a Si substrate; (b) PLD of thin Ni film on the Si substrate covered with WS_2_; and (c) PLD of thin g-C film on the Si substrate covered with bilayer Ni/WS_2_ film. To obtain multilayer WS_2_/g-C/Ni/WS_2_ coating, RPLD of thin WS_2_ film was applied again. Figure S2: SEM image and EDS surface distribution of different elements in the WS_2_(C, Ni) thin film coating deposited on Si substrate. The distribution of W Ma1 is not shown because it was identical to the distribution of silicon. Quantification of tungsten in the presence of silicon is difficult due to the overlap of the W Ma1 peak with the Si Ka1 of the substrate. The figure illustrates the presence of Ni particles of micron, submicron, and nanometer sizes. Carbon rather effectively covers Ni particles of submicron and nanometer sizes; however, Ni particles with a size close to a micron are not uniformly covered with carbon. This is probably responsible for its modification during RPLD of the WS_2_ in H_2_S film. An increase in the intensity of Ka1 peak around Ni particle (located at ~3−4 mm) indicates the possibility of sulfurization of the Ni microparticle. Figure S3: (a) MRS spectrum for g-C layer; (b) cross-section TEM image of the interface layer in WS_2_(C, Ni) thin-film coating. The structure of the WS_2_/g-C interface layer was studied by high-resolution transmission electron microscopy (HRTEM), using a Carl Zeiss Libra 200FE microscope. The HRTEM image confirms the formation of layered 2H-WS_2_ film on the disordered surface of g-C layer. Figure S4: SEM image and EDS spectra for different regions in the wear track for WS_2_(C) thin-film coating. Figure S5: SEM image and EDS-measured surface distribution of different elements in the WS_2_(C) thin-film coating across the wear track. Figure S6: MRS spectrum of WS_2_(C, Ni) thin-film coating, which was measured with increased laser intensity for the wear debris located at the end of the wear track. Figure S7: SEM image and EDS spectra for different regions in the wear track for WS_2_(C, Ni) thin-film coating. Figure S8: SEM image and EDS-measured surface distribution of different elements in the WS_2_(C, Ni) thin-film coating around the S-enriched area in the wear track.
