# Supplementary material for: Tribological Properties of WS2 Thin Films Containing Graphite-like Carbon and Ni Interlayers
Source: Materials (Basel). 2022 Dec 28;16(1):282. doi: 10.3390/ma16010282 (PMC9822394; doi:10.3390/ma16010282)
Supplement: Supplementary file 1 [file materials-16-00282-s001.zip › materials-2077576-supplementary.pdf]

Supplementary Materials

Tribological properties of WS_2 thin films containing graphite-like carbon and Ni interlayers

Roman I. Romanov[1], Dmitry V. Fominski[1], Maxim V. Demin[2], Mariya D. Gritskevich[1], Natalia V. Doroshina[3], Valentyn S. Volkov[3], and Vyacheslav Yu. Fominski[1,*]

[1] National Research Nuclear University MEPhI (Moscow Engineering Physics Institute), Moscow, Kashirskoe sh., 31, Russia, 115409
[2] Immanuel Kant Baltic Federal University, Kaliningrad, A. Nevskogo St 14, Russia, 236016
[3] Center for Photonics and 2D Materials, Moscow Institute of Physics and Technology (MIPT), Dolgoprudny, Russia, 141701
* Correspondence: vyfominskij@mephi.ru

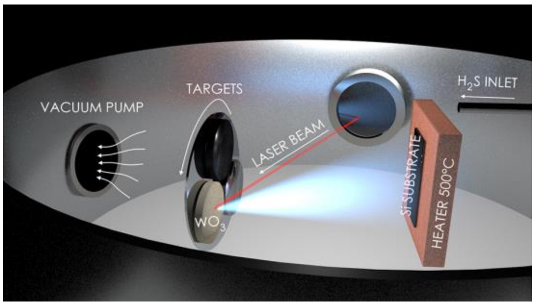

(**a**)

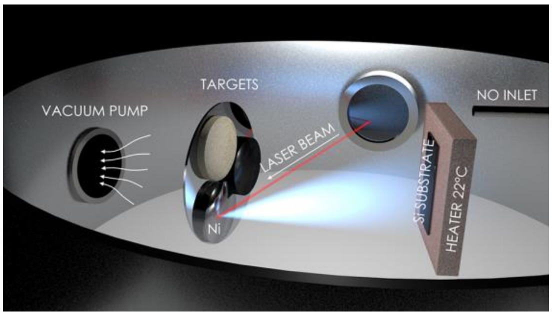

(**b**)

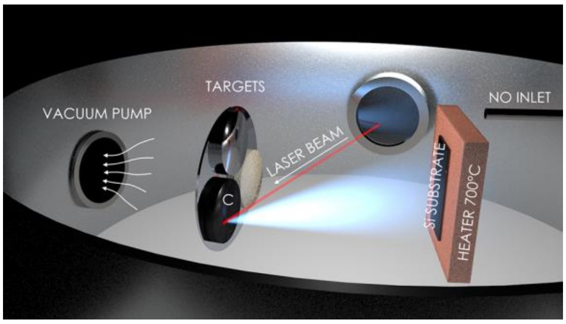

(**c**)

Figure S1: A scheme of fabrication of multilayer thin-film coatings using the laser technique: (a) RPLD of thin WS_2 film on a Si substrate; (b) PLD of thin Ni film on the Si substrate covered with WS_2; (c) PLD of thin g-C film on the Si substrate covered with bilayer Ni/WS_2 film. To obtain multilayer WS_2/g-C/Ni/WS_2 coating, RPLD of thin WS_2 film was applied again.

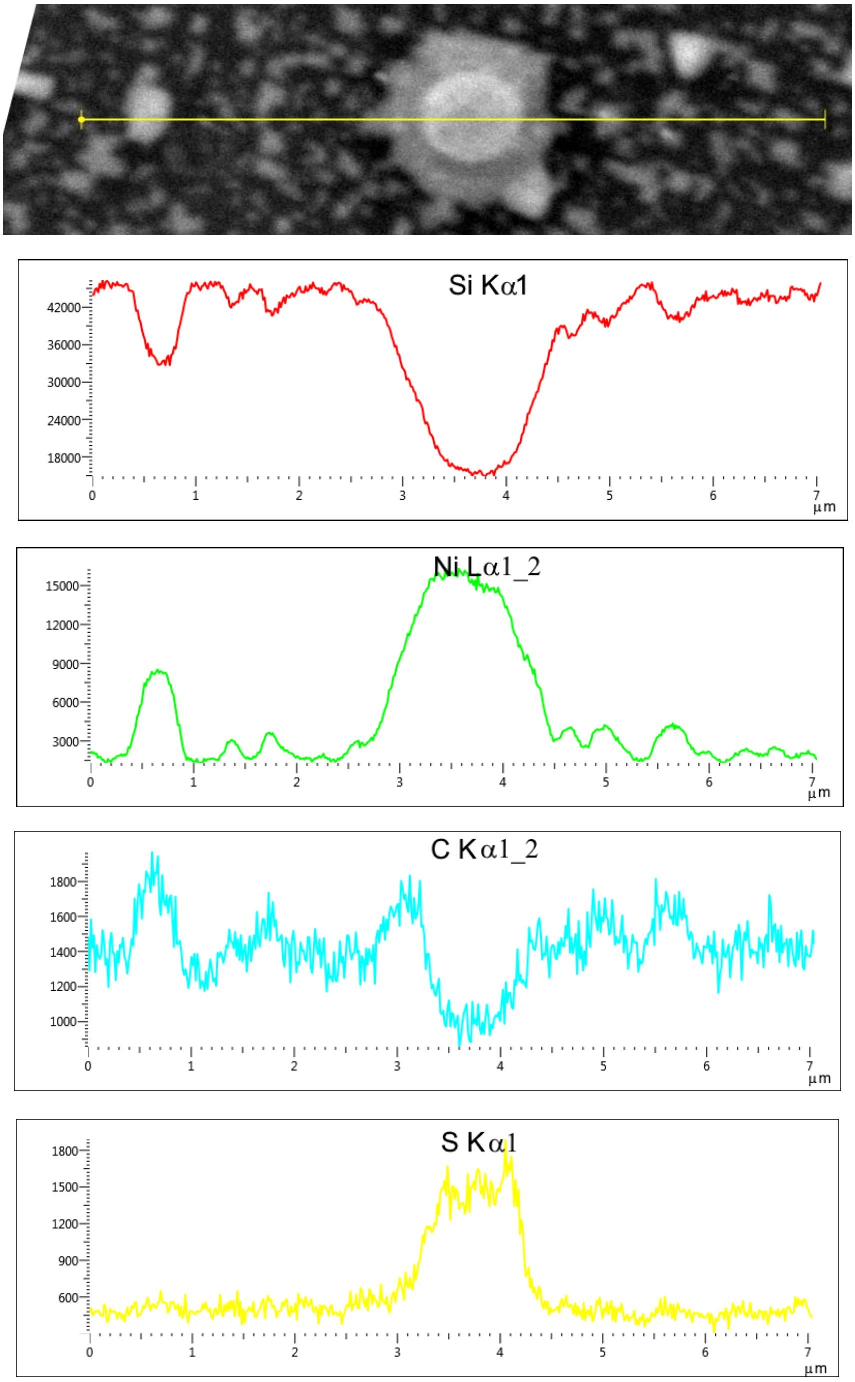

Figure S2. SEM image and EDS surface distribution of different elements in the WS_2(C,Ni) thin film coating deposited on Si substrate.

The distribution of W Ma1 is not shown because it was identical to the distribution of silicon.

Quantification of tungsten in the presence of silicon is difficult due to the overlap of the W Ma1 peak with the Si Ka1 of the substrate.

The figure illustrates the presence of Ni particles of micron, submicron, and nanometer sizes. Carbon rather effectively covers Ni particles of submicron and nanometer sizes, however, Ni particles with a size close to a micron are not uniformly covered with carbon. This is probably responsible for its modification during RPLD of the WS_2 in H_2S film. An increase in the intensity of Ka1 peak around Ni particle (located at ~3–4 mm) indicates the possibility of sulfurization of the Ni microparticle.

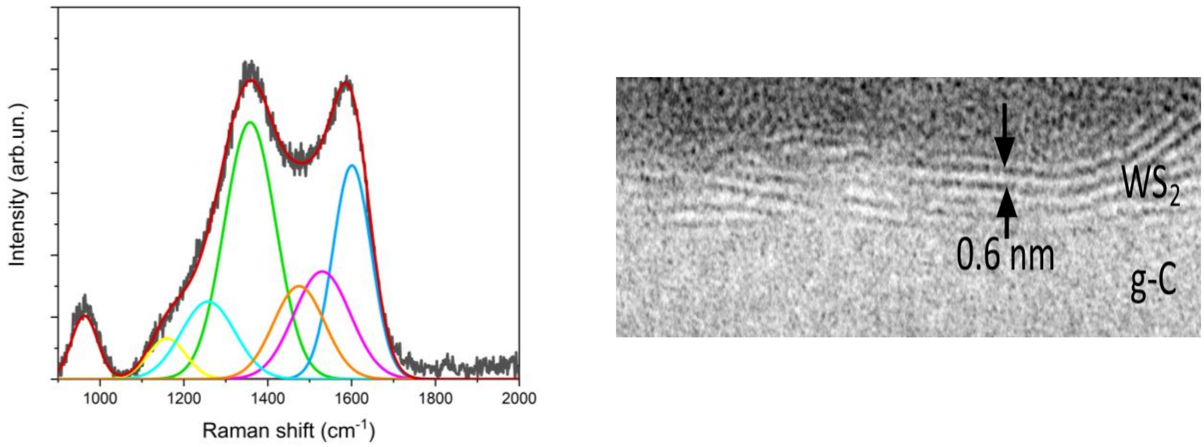

(a) (b)

Figure S3. (**a**) MRS spectrum for g-C layer; (**b**) cross-section TEM image of the interface layer in WS_2(C,Ni) thin-film coating. The structure of the WS_2/g-C interface layer was studied by high-resolution transmission electron microscopy (HRTEM), using a Carl Zeiss Libra 200FE microscope. The HRTEM image confirms the formation of layered 2H-WS_2 film on the disordered surface of g-C layer.

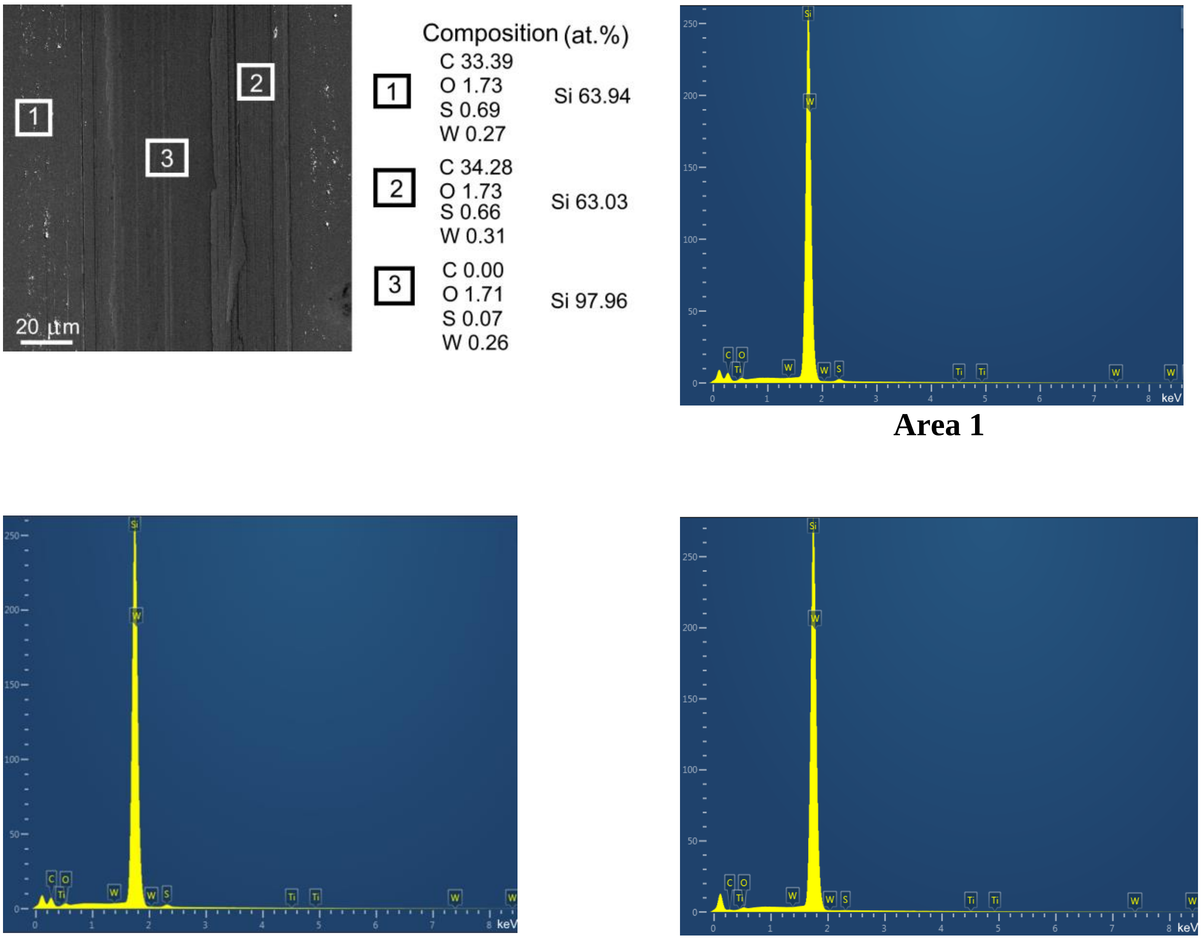

Area 1

Area 2

Area 3

Figure S4. SEM image and EDS spectra for different regions in the wear track for WS_2(C) thin-film coating on Si substrate.

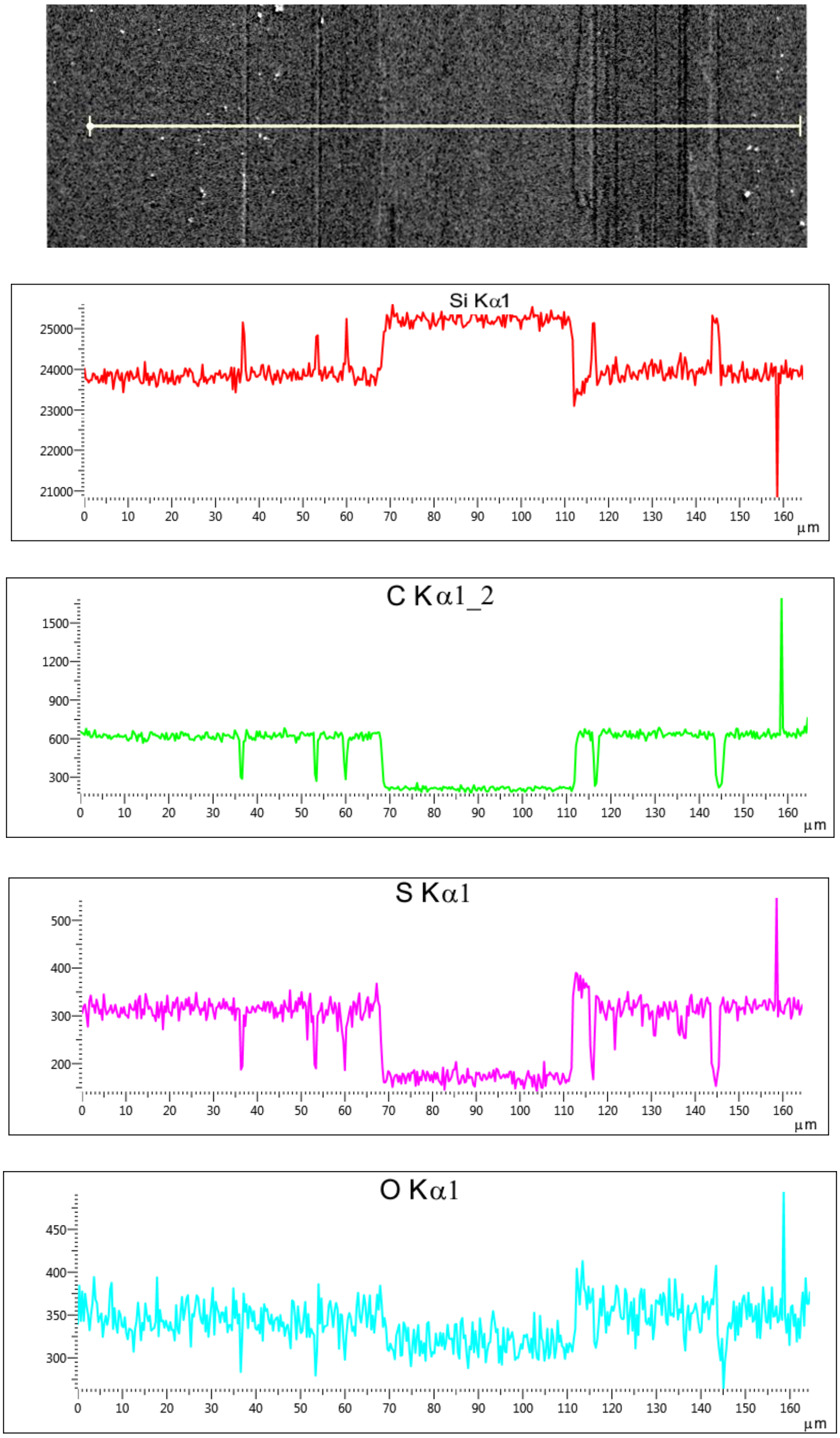

Figure S5. SEM image and EDS-measured surface distribution of different elements in the WS_2(C) thin-film coating across the wear track.

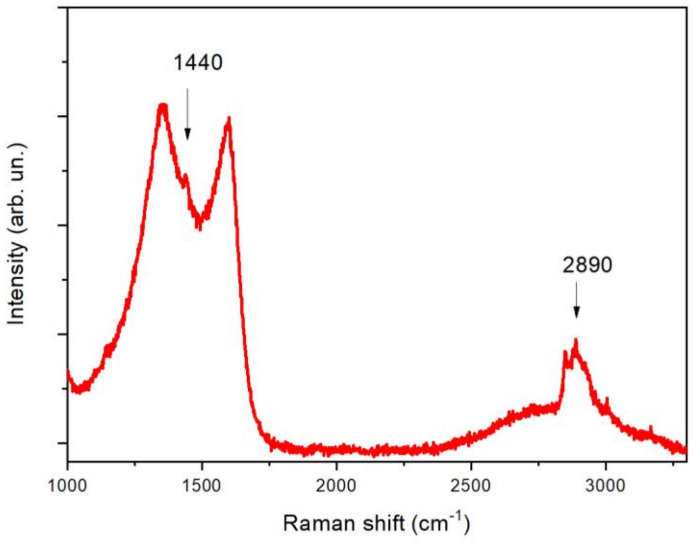

Figure S6. MRS spectrum of WS_2(C,Ni) thin-film coating which was measured with increased laser intensity for the wear debris located at the end of the wear track.

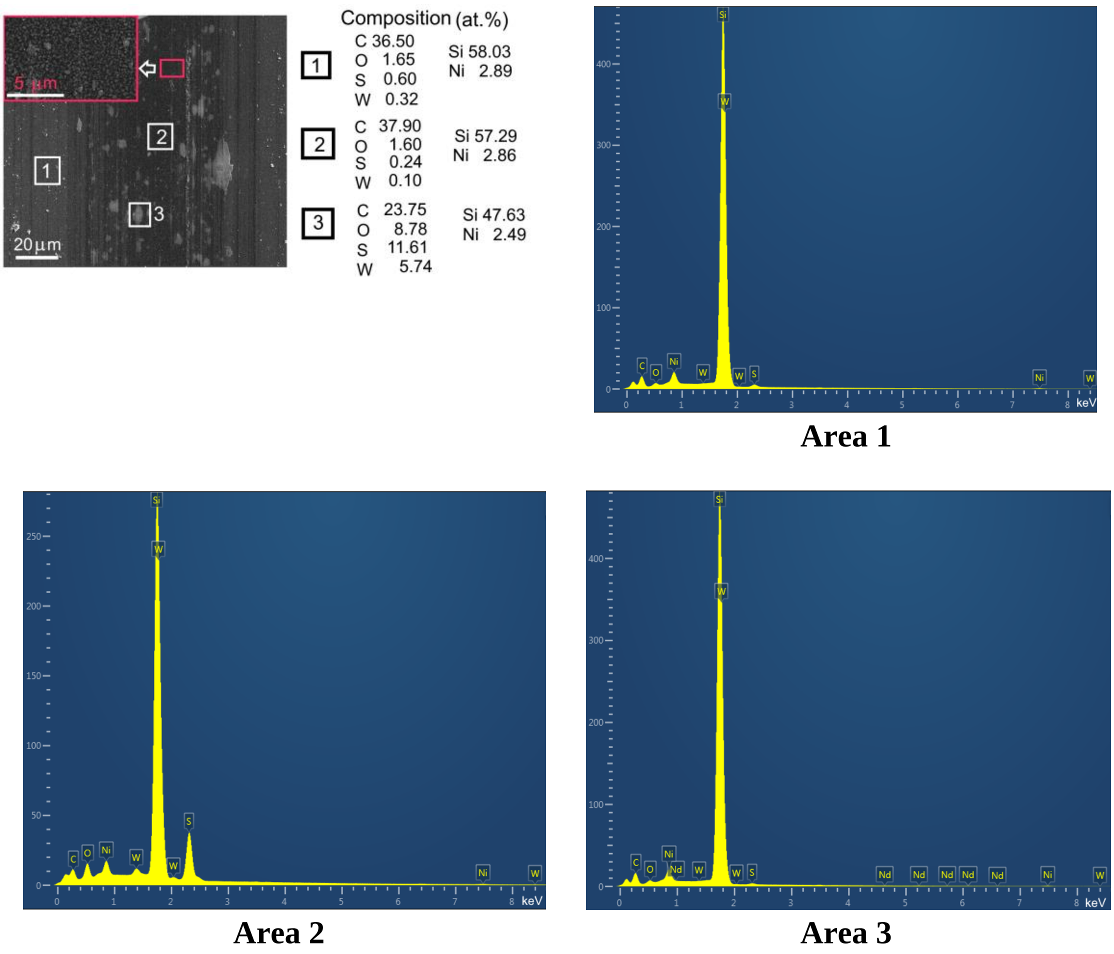

Figure S7. SEM image and EDS spectra for different regions in the wear track for WS_2(C,Ni) thin-film coating.

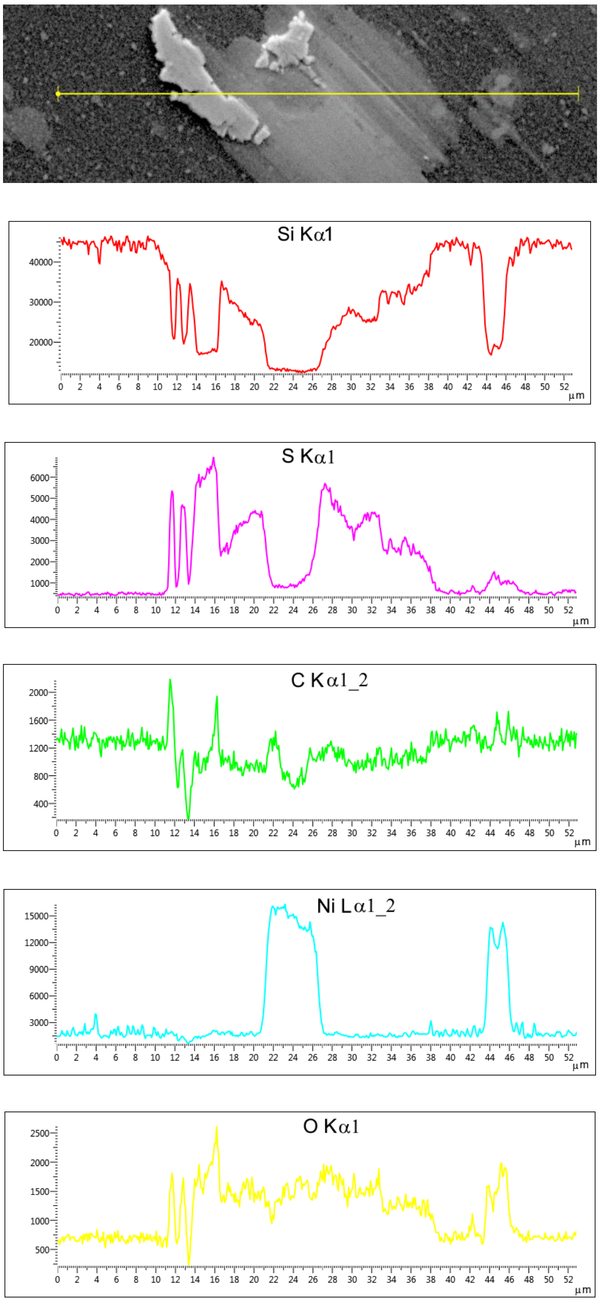

Figure S8. SEM image and EDS-measured surface distribution of different elements in the WS_2(C,Ni) thin-film coating around the S-enriched area in the wear track.
